# An international survey on hybrid imaging: do technology advances preempt our training and education efforts?

**DOI:** 10.1186/s40644-018-0148-6

**Published:** 2018-04-26

**Authors:** T. Beyer, R. Hicks, C. Brun, G. Antoch, L. S. Freudenberg

**Affiliations:** 1 QIMP Group, Centre Medical Physics and Biomedical Engineering, Medical University Vienna, Währinger Str 18-20/4L, 1090 Vienna, Austria; 2 The Sir Peter MacCallum Department of Oncology, the University of Melbourne, Melbourne, 3000 Australia; 3 European Society for Hybrid Medical Imaging, Neutorgasse 10, 1010 Vienna, Austria; 4 Department of Diagnostic and Interventional Radiology, University Düsseldorf, Medical Faculty, Moorenstrasse 5, 40225 Düsseldorf, Germany; 5 ZRN Rheinland, Ueberseite 88, 41352 Korschenbroich, Germany

**Keywords:** Hybrid imaging, Training and education, Specialization, Conflict, Profession

## Abstract

**Background:**

Hybrid PET/CT and PET/MRI are increasingly important technologies in the evaluation of malignancy and require cooperation between radiologists and specialists in molecular imaging. The aim of our study was to probe the mindsets of radiological and nuclear medicine professionals in regard to current hybrid imaging practice and to assess relevant training aspirations and perceived shortfalls, particularly amongst young professionals. In this context, we initiated an international survey on “Hybrid Imaging Training”.

**Methods:**

An online survey was prepared on-line and launched on October-2, 2016. It was composed of 17 multiple-choice and open questions regarding the professional background, a perspective on hybrid imaging training efforts and lessons to be learned from disparate craft groups. The survey ran for 2 weeks. We report total responses per category and individual free-text responses.

**Results:**

In total, 248 responses were collected with a mean age of all responders of (41 ± 11) y. Overall, 36% were within the target age range of (20–35) y. Across all responders, the majority (72%) commented on there being too few hybrid imaging experts in their country, whereas only 1% said that there were too many. Three quarters of the responders were in favour of a curriculum allowing sub-specialisation in hybrid imaging. With respect to reporting of hybrid imaging, confidence increased with age. The average rating across all responders on the level of cooperation among the two specialties suggested a low overall level of satisfaction. However, the survey feedback indicated the local (on-site) cooperation being somewhat better than the perceived cooperation between the relevant associations on a European level.

**Conclusion:**

We consider these results to represent an appropriate cross-section of professional opinions of imaging experts across different demographic and hierarchical levels. Collectively they provide evidence supporting a need to address current shortfalls in developing hybrid imaging expertise through national educational plans, and, thus, contribute to helping improve patient care.

## Background

Over the centuries, there has been continuous improvement in the diagnosis and treatment of diseases. However, cardiovascular, cancerous and neurodegenerative diseases still pose a major challenge to healthcare systems today [1]. Patients suffering from any of these diseases expect an accurate diagnosis, which in almost all cases will be guided by non-invasive imaging procedures. These may include, for example, Computed Tomography (CT) and Magnetic Resonance Imaging (MRI) to assess the anatomy and morphological alterations of patients with great visual detail and spatial resolution [2], or Single Photon Emission Computed Tomography (SPECT) and Positron Emission Tomography (PET) that permit the assessment of metabolic and signaling pathways and their variants [3]. All of these imaging modalities represent exciting instrumentational and methodological approaches to diagnosing patients, and help us understand diseases better [4].

Stand-alone imaging modalities were first conceived in the 1950’s and 1960’s and have entered the market about a decade later. Today, there are ten thousand’s of these imaging systems operational worldwide. While each modality has its merits it became clear that a combined use of “anatometabolic” imaging [5] had the potential to improve the diagnostic value of non-invasive imaging, and, thus, benefit patients and healthcare systems alike. As a consequence, we have witnessed the introduction of combined SPECT/CT [6], PET/CT [7] and PET/MR systems [8] in the first decade of the twenty-first century. Although the adoption of these hybrid imaging systems has varied widely over time and across regions, their clinical traction has grown and proven to yield a diagnostic benefit in a variety of clinical questions [9–11].

Whole-body PET/CT was introduced in 2001 with multiple generations and updates of PET/CT following suit; however, an international survey performed in 2011 demonstrated that the majority of PET/CT users still disregarded the diagnostic power of CT in a large fraction of patients [12]. Likewise, a survey performed in 2014 among international SPECT/CT users demonstrated that an overwhelming majority wanted to employ the SPECT/CT as a SPECT system with attenuation correction and coarse anatomical localization rather than assuming a closer integration of radiology-driven imaging perspectives [13]. When combined PET/MR imaging of humans was introduced a decade ago, an overwhelming number of studies focused (again) on attenuation correction [14] and a potential diagnostic equivalence of DIXON-type MR and low-dose CT images for the anatomical localization of the PET findings [15]. These are but a few examples that attest to a continuous hesitation towards a game-changing adoption of hybrid imaging and, in turn, to an ongoing debate about key responsibilities and ownership issues in hybrid imaging. This is particularly disconcerting in the field of cancer imaging since hybrid imaging techniques, such as [^99m^Tc]MDP-SPECT/CT and [^18^F]FDG-PET/CT, are widely used for the staging of common malignancies. With increasing availability of more specific tracers for various types of cancer, the penetration of these technologies into oncological imaging is likely to further increase [16]. An example of this is the use of [^68^Ga]PSMA imaging for prostate cancer, which is likely to impact both PET/CT and PET/MRI imaging [17].

The territorial approaches towards the clinical adoption of “anatometabolic imaging” [5] originate to a large extent from perspectives of people and professionals whose experience and model of practice were developed prior to the availability of hybrid imaging [18]. However, to date, we have no information about the mindset of “early” professionals in regard to combined imaging and their perceptions of relevant training requirements. This especially includes the new generation of young professionals who have been exposed only to a period of merging technologies and practices. Therefore, we have set out to probe the opinions on hybrid imaging of, in particular, the next generation of imaging specialists and to compare them to senior clinicians.

## Methods

We have initiated an international survey, entitled “Hybrid Imaging Training” following the organization of the 3rd International Hybrid Imaging Course [19]. The survey consisted of 17 questions regarding the demographics and professional background of the responders (6), their confidence in dealing with hybrid imaging and their perspective on hybrid imaging training efforts (6), as well as lessons to be learned from disparate craft groups (5) (Table 1). In order to better understand variations in cross-specialty appreciation, we queried also what “nuclear medicine” could learn from “radiology”, and vice versa (Table 1).

**Table 1:** Hybrid Imaging Survey with 17 questions geared towards the probing the knowledge and attitude of young healthcare professionals in the context of clinical hybrid imaging

Demographics	
Q1	Your age (y) and gender (m, f)
Q2	Your country of employment
Q3	Your place of work: radiology, nuc, joint common rad-nuc, nuc as part of rad, other
Q4	Who is the head of your department (if any): rad, nuc, physics, other ...?
Q5	Are you an MD w/ or w/o board certification (Nuc, Rad, Nuc/Rad, other)?
Q6	How many years of hybrid imaging experience do you have (y): SPECT/CT, PET/CT, PET/MR?
Training	
Q7	In general, how confident do you feel when reporting hybrid imaging studies today (scale 1–10)?
Q8	What type of continuous education means (hybrid imaging) do you employ for yourself: literature, on-line courses/webinars, conferences, special courses (inter−/national), or fellowships (multiple choice, and each option ir−/regularly)?
Q9	In your country of employment, are there sufficient hybrid imaging experts (too few, too many, just right, don’t know)?
Q10	Would you like a curriculum for a subspecialization for a certified, hybrid imaging expert (y, n, don’t know)?
Q11	Would you like a joint curriculum (common trunk residency, or alike) for radiology and nuclear medicine (y/n)?
Q12	Are you aware of the Joint White Paper of ESR and EANM (https://www.ncbi.nlm.nih.gov/pubmed/17609961) (y/n)?
Cross-fertilization	
Q13	From your perspective, how well do the European Society of Radiology (ESR) and the European Association of Nuclear Medicine (EANM) work together (scale 1–10)?
Q14	From your perspective, how well do your national Associations for Radiology and Nuclear Medicine work together (scale 1–10)?
Q15	From your personal experience, how well do radiologists and nuclear medicine specialists work together at your local hospital/institution/site (scale 1–10)?
Q16	What would you like to see happening in the next 5 years with regards to hybrid imaging (free text)?
Q17	In your opinion, what could radiology learn from nuclear medicine (free text) and what could nuclear medicine learn from radiology (free text)?

The survey was composed via Google Documents and a link was mailed to all participants of the 2016 and 2017 Asklepios ESOR (European School of Radiology) and ESHI (European Society for Hybrid Medical Imaging) Courses on Hybrid Imaging. Furthermore, it was advocated through the Aunt Minnie community on Oct-2, 2017, in addition to numerous individual mailings within the professional networks of the co-authors. Anonymized responses were received from Oct-2 to Oct-16, 2017 and tabulated for each question.

We report total responses per category; minimum, and maximum values (when applicable). The individual free-text responses were analysed using a content analysis that permits the inclusion of textual information and the systematic identification of properties, such as the frequencies of the most frequently used keywords by locating the most important structures of its communication content [20].

## Results

In total, 248 eligible responses were collected. Of those, 149 and 164 eligible free text responses were received for the free text question on the cross-specialty learning experience for nuclear medicine and radiology, respectively.

### Demographics

**Fig. 1:**
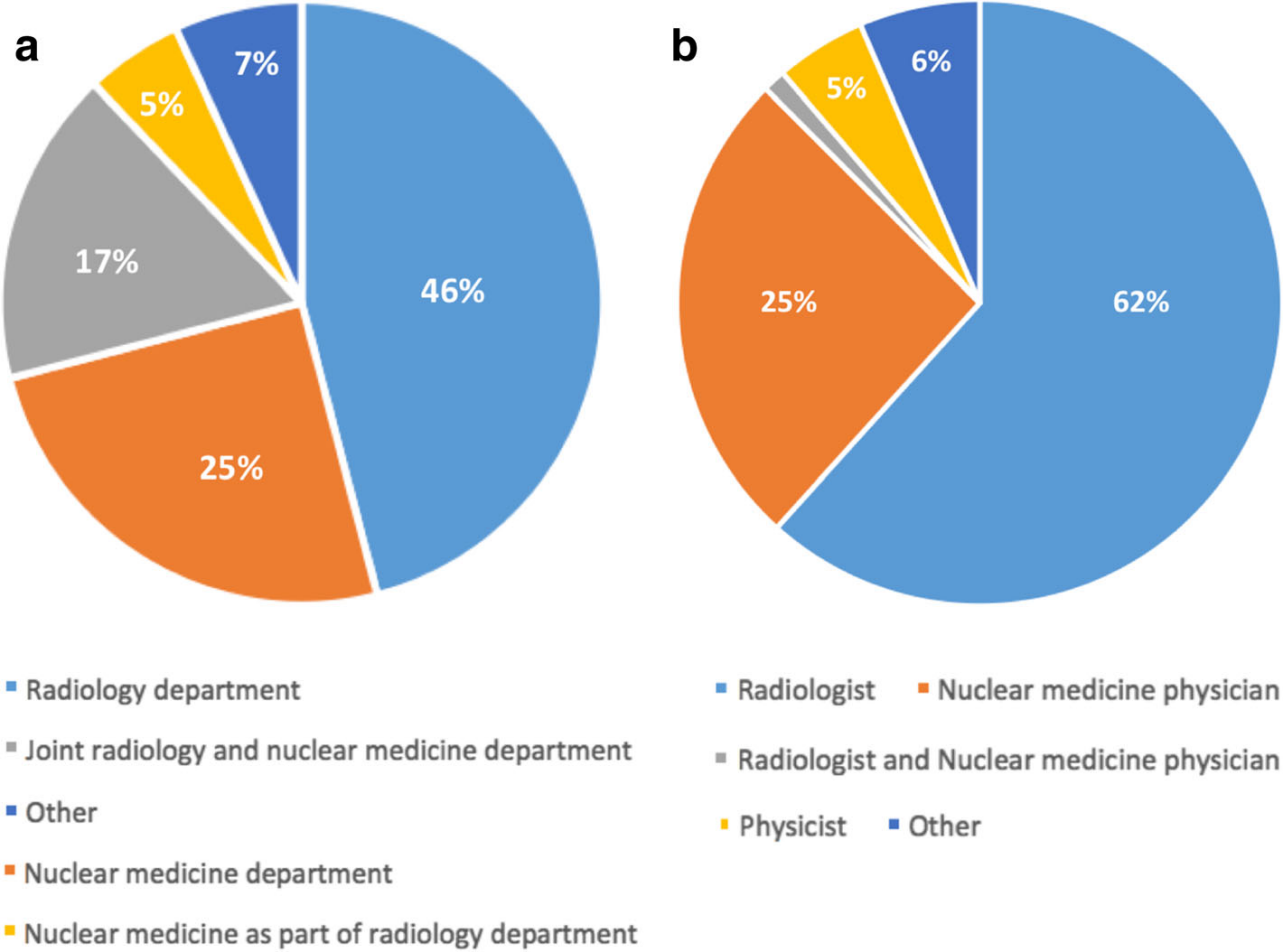
**a** Place of work of the responders. **b** Head of the department where responders work at

The mean age of all responders was (41 ± 11) years with 36% being of age 20-y to 35-y, and with 4% being older than 65-y. The majority of responders were male (65%). The highest number of responses (197/248) were collected from Europe (78%), followed by Africa (6%), Asia (6%), Middle East (4%), North Americas (4%) and South America (2%). Half of the responders work in Radiology departments (51%), 25% work in Nuclear Medicine departments, 17% in joint Radiology and Nuclear Medicine departments, and 7% in other institutions (Fig. 1a). Most departments, presumably including the joint departments, are run by a radiologist (62%), while one fourth is operated by a nuclear medicine physician (Fig. 1b). Across all responders, 18% were without any board certification, 36% and 18% were certified in radiology and nuclear medicine, respectively, while 15% were dual-certified. A third of the responders indicated that they had no experience with hybrid imaging, 22% said they had up to 4 years of experience with hybrid imaging and 22% indicated more than 10 years of experience.

### Training

**Fig. 2:**
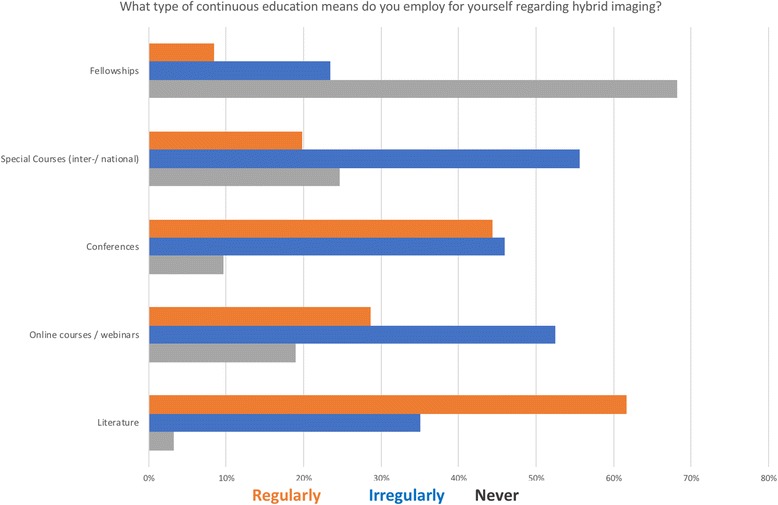
Frequency (ir/regularly or never) of use of various training and education options across all 248 responses

Report confidence increased with age (and experience) but less than half the responders (43%) indicated they were very confident (score 8–10 on a scale from 1 to 10) in reporting hybrid images. When being asked about their personal engagement in various education and training options, it became obvious that more accessible educational material increased its use (Fig. 2). When analyzing educational endeavours per age category, professionals without a board certification engage regularly in reviewing scientific literature (63%) and conference attendance (48%) with a comparatively low regular interest (27%) in webinars.

Across all responders, the majority (72%) commented on too few hybrid imaging experts being available in their country; 16% said there are just as many experts as needed, while 1% said there were too many. Responses were very similar if considering responders from Europe only (72%, 15% and 1%).

Three-quarters of the responders were in favour of a curriculum for a subspecialisation as a hybrid imaging expert (75% worldwide versus 73% in Europe). Most opponents were radiologists by training. Close to 90% of the responders were in favour of a joint curriculum along the suggested training options laid out in the previous White Paper developed jointly by the European Society of Radiology (ESR) and the European Association of Nuclear Medicine (EANM) [21].

### Cross-fertilization

**Fig. 3:**
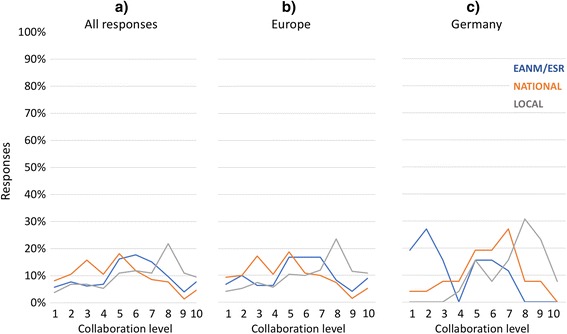
Quality of collaboration (1-very low, 10-very high) of radiologists and nuclear medicine specialists on a local (on-site), national and European level: (**a**) all 248 responses, (**b**) European responses only, and (**c**) responses from Germany only

Figure 3a illustrates the average rating across all responders of the level of cooperation (1-very low, 10-very high) among the two specialties on a local, a national and a European basis showing that the overall level of satisfaction is low. The same observation was made across responses from European countries only (Fig. 3b). However, the survey feedback suggests the local (on-site) cooperation being somewhat better than the perception of cooperation between the relevant associations, EANM and ESR. To take this further, we analyzed the level of cooperation in Germany, where the cooperation of the national societies, the German Society of Nuclear Medicine - DGN and the German Society of Radiology - DRG, was perceived much better than that on the European level, with the same observation being made for the collaboration on a local level (Fig. 3c).

#### What can nuclear medicine learn from radiology?

The majority of the responders gave priority to “anatomy” (62%), followed by “competence in CT” (11%) and “competence in MRI” (12%), as well as knowledge of “comprehensive radiological imaging” (7%). Of note, many comments (> 20%) suggested that nuclear medicine should learn about “efficacy”, “dynamics”, “patient throughput and management”, and “standardization”. Responders also frequently suggested that nuclear medicine should learn “confidence” from their radiology counterparts. No differences between the younger (up to 35 years) and older participants (older than 35 years) were found. Some examples of individual responses included:

- “Nuclear medicine could learn precise localization of a lesion and structural changes in a disease” (from a 44-y/o male Austrian radiologist)
- “Dynamics. Radiology, particularly MRI, is a dynamic field and new techniques are currently being developed for clinical applications. Radiologists - I find - are more open to change and adapt quickly. Nucs can be a bit stiff to change.” (from a 35-y/o female Canadian fellow in Radiology)
- “Patho-/anatomy, clinical integration and acceptance in clinical context, imaging technology, synoptical overviews as to what imaging method is best for which question.” (from a 53-y/o male Swiss dual board certified medical doctor).

#### What radiology can learn from nuclear medicine?

Most responders stated that nuclear medicine teaching points for radiologists include “metabolic imaging” (49%) and the “knowledge of function, physiology and pathophysiology” (27%). Other important learning objectives included “radiopharmacy” (17%), quantification (6%), and knowledge of “comprehensive nuclear medicine imaging” (7%). Beyond that, many comments (> 25%) suggested that radiology should learn from nuclear medicine about “being physicians”, “cooperation”, “respect”, “self-criticism”, and “different way of thinking”. No differences between the younger (up to 35 years) and older participants (older than 35 years) were found. Some specific responses were:

- “Nuclear Medicine imaging is a more physiologic way of image interpretation than an anatomic.” (from a 41-y/o male Dutch nuclear medicine physician)
- “That life isn’t black and white. Applied physiology. Therapeutic approach.” (from a 48-y/o male German nuclear medicine physician)
- “Modesty.” (from a 44-y/o male German dual board certified medical doctor)
- “Quality control: Nucs are used to reading images looking for potential artefacts in the image. […] They don’t blindly trust the images. They are aware of the limitations of their imaging modality and can interpret images even in the presence of some noise. They are I would say, well trained Support Vector Machines.” (from a 35-y/o female Canadian fellow in radiology)

#### Other comments offered by the participants

Of note, 19 participants took the opportunity not only to answer both questions but to comment on the relationship between nuclear medicine and radiology. Some stress the obvious differences; others state that cooperation or common training are key. There were no distinguished differences between older and younger participants. Some specific comments were:

- “They [Nuclear Medicine and Radiology] are not separated. They complete each other in imaging diagnostic field.” (from a 27-y/o female Albanian radiologist)
- “I think it’s compatible and complementary.” (from a 33-y/o female Polish fellow in radiology)
- “[One should] wash out the borders between radiology and nuclear medicine territories, rise up new specialisation - imaging experts with organ-specific sub-specialisations.” (from a 37-y/o female Italian nuclear medicine physician)
- “Combination is the future solution.” (from a 54-y/o female Swedish dual board certified medical doctor)
- “[Nuclear medicine] is a whole new discipline with a different way of thinking.” (from a 64-y/o female Hungarian nuclear medicine physician)
- “My personal view is that I never learn anything more from NM specialist as the metabolic information is something as an additional quality of image as very well-known from multi-parametric imaging.” (from a 47-y/o male Czech radiologist)

## Discussion

The aim of this survey was to get an insight into current professional perspectives on the practice of hybrid imaging and any pertinent need for training and educational strategies for the international adoption of clinical hybrid imaging. Although this survey was geared towards collecting a global feedback, most of the responses (78%) collected were from Europe, thus, rendering this data more Eurocentric; similarly, most of the responders were male (65%). Hence, we assume that the heterogeneity of answers we described will be even more manifold.

### Training

Taken together, the data above show that the majority of medical professionals in the domains of radiology and nuclear medicine want to engage in continuous education. Furthermore, there is a persistent interest and willingness in joint training programs. When being asked about their personal adoption of various education and training options (e.g., literature, on-line courses, attending conferences or special courses and fellowships), it became obvious that the more accessible are the educational offers, the more frequently they are being adopted; this refers to accessing literature while fellowships, for example, are rarely used. On-line courses and webinars are used infrequently, and there is room for more engagement by authorities and specialist’s assemblies, particularly, In view of limited funds available.

### Collaboration of nuclear medicine and radiology - theory (white paper)

Our survey indicated also that over half (54%) of responders were unaware of the joint ESR and EANM statement from 2007 [21, 22]. People who knew about this white paper were generally older than those who did not (46-y vs 38-y); likewise, they were mainly nuclear medicine specialists rather than radiologists (31% vs 19%). When being asked about a joint curriculum along the suggested training options laid out in the White Paper [21], 87% responded with “yes”, with the fraction of proponents growing somewhat larger with professional age. Both responses are in line with the survey conducted among EANM and ESR members in 2010, when 77% and 85%, respectively supported the idea of an interdisciplinary training programme [23].

### Collaboration of nuclear medicine and radiology – Practice

The White Paper closed by stating “Both organizations [ESR and EANM] are committed to working together for the future benefit of both specialties” [21, 22]; our data indicate that a decade into hybrid imaging, this statement is perceived to have not yet materialized. The opinion trend surveyed here indicates a need for cooperation between the two specialties and a persistent wish for a strategy towards integrating hybrid imaging expertise into an interdisciplinary training [23–25], or into alternative forms of restructured training modules to account for multi-modality imaging [26].

As early adopters of hybrid imaging, we suggest embracing the numerous opportunities of hybrid imaging for the benefit of clinical patient management and healthcare systems. We should seize the opportunities of presenting high-sensitivity molecular information in judiciously tailored anatomical and morphological reference frames to engage referring clinicians in fostering personalized treatment plans, and to engage with other medical specialties in an attempt to merge knowledge about diseases for building models that help predict and assess disease of other patients in the future; big data can get bigger with hybrid images.

Our survey shows variations of the collaborations between the two specialties on local, national and international levels. Most participants consider the professional collaboration on a local level more positive than the collaboration between the National and European associations. In our view, this is an indicator that cooperation grows more complicated as soon as politics get involved. An interesting divergent finding is the considerably well-perceived cooperation of the Societies of Radiology and Nuclear Medicine in Germany (Fig. 3c). They have a long history of joint efforts towards the adoption of dual-modality imaging, given the national funding scheme for imaging system acquisitions put forward by the National Research Foundation (DFG), which forces the specialties to cooperate. Also, Germany has established a potential role model in negotiating joint training programs, as attested by the ongoing reviews of the national continuous educational procedures for radiology [27] and nuclear medicine [28].

Naturally, the engagement of local and national stakeholders takes time, but eventually helps build a sustainable framework for the continuous and efficient adoption of new imaging technologies without jeopardizing the core expertise of the adjoined specialties. Our survey indicates a wish of the daily users, including the next generation of “anatometabolic imagers”, to extending similar collaborative efforts to the National and European level (Fig. 3a, b).

### Beyond cooperation: What can we learn from each other?

The results of our content analysis above appear to reinforce the concept of “habitus” [29] as introduced by Pierre Bourdieau [30], who described the habitus as “a system of embodied dispositions, tendencies that organize the ways in which individuals perceive the social world around them and react to it. These dispositions are usually shared by people with similar background […] and reflect the lived reality to which individuals are socialized, their individual experience and objective opportunities. Thus, the habitus represents the way group culture and personal history shape the body and the mind, and, as a result, shape social action in the present.” Our survey (Table 1) suggests that the fundamental difference of a focus on physiology or anatomy led to different habitus’ in the two specialist fields with respect to imaging, patient care, and self-perception. One participant even stated in short: “Nuclear medicine physicians are better physicians; radiologists are better imagers.”

Perhaps this type of statement in the light of clinical hybrid imaging lends itself to the definition of a new mindset for hybrid imagers, which could be addressed from the start through a more intense collaboration, or, even better and more sustainable, a joint training and education path that helps bridge the differing mindsets of nuclear medicine physicians and radiologists, for they both do care about their patients. Such training path could start with the integration of radiologists and nuclear medicine physicians as members of a local multi-disciplinary clinical team, pending the initiation of a new or the continuous expansion of existing communication platforms involving the two specialties. Further, both specialties could establish disease-centric fellowships that include subspecialty hybrid imaging training, such as a Cancer Imaging fellowship as to equip the younger generation with the tools and knowledge needed to demonstrate the impact that imaging can have on patient management and outcome (e.g., http://www.esor.org/cms/website.php?id=/en/programmes/exchange_programmes_for_fellowships.htm). And finally, training efforts could be formalized in residency programmes, including a dedicated cancer imaging curriculum, such as that advocated by Howard and colleagues [31].

We appreciate that web-based surveys have drawbacks, such as lower response rates compared to other survey modes [32]. Nonetheless, we decided to benefit from the easy, rapid, and widespread distribution of Web-based questionnaires. Furthermore, Web-based surveys offer logistic advantages such as fast response collection and low costs [33]. As such, we consider these results a representative cross-section of professional opinions by imaging experts across different demographic and hierarchical levels that may help contribute to recognizing a need to better address needs for hybrid imaging expertise in national educational plans, and, thus, contribute to helping improve patient care. We hope this knowledge may help to refocus the discussions about the “homeland” of hybrid imaging from professional politics back to patient care. In short: hybrid imaging should be performed to the best possible diagnostic quality standards with the patient in mind - no more, no less.

## Conclusion

Our international field study of hybrid imaging adopters indicates a persistent interest, particularly of the younger generation of imaging professionals, to offer training programmes to support the education and certification of hybrid imaging experts. Free text interviews yield valuable insights into the professional vanities of radiology and nuclear medicine experts, but can help define learning objectives in joint curricula. Cancer imaging is a field in which hybrid technologies already have an important role, and this is likely to expand. Therefore, the oncological imaging community could take a lead in improving training programmes and harmonising reporting methodology for the sake of a sustainable adoption of hybrid imaging techniques.

## References

[1] http://www.who.int/mediacentre/factsheets/fs310/en/.

[2] Brant W, Helms C, Brant W, Helms C (2007). Fundamentals of diagnostic radiology.

[3] Valk PE (2003). Positron emission tomography. Basic science and clinical practice.

[4] James ML, Gambhir SS (2012). A molecular imaging primer: modalities, imaging agents, and applications. Physiol Rev.

[5] Wahl RL (1993). “Anatometabolic” tumor imaging: fusion of FDG PET with CT or MRI to localize foci of increased activity. J Nucl Med.

[6] Patton JA, Delbeke D, Sandler MP (2000). Image fusion using an integrated, dual-head coincidence camera with X-ray tube-based attenuation maps. J Nucl Med.

[7] Beyer T (2000). A combined PET/CT tomograph for clinical oncology. J Nucl Med.

[8] Schlemmer H (2008). Simultaneous MR/PET imaging of the human brain: feasibility study. Radiology.

[9] Bockisch A (2009). Hybrid imaging by SPECT/CT and PET/CT: proven outcomes in cancer imaging. Semin Nucl Med.

[10] Beyer T, Veit-Haibach P (2014). State-of-the-art SPECT/CT: technology, methodology and applications-defining a new role for an undervalued multimodality imaging technique. Eur J Nucl Med Mol Imaging.

[11] Bailey D (2017). *Combined PET/MRI: Global Warming-Summary Report of the 6th International Workshop on PET/MRI, March 27–29,* 2017.

[12] Beyer T, Czernin J, Freudenberg L (2011). Variations in clinical PET/CT operations: results from an international survey among active PET/CT users. J Nucl Med.

[13] Wieder H (2012). Variations of clinical SPECT/CT operations: an international survey. Nuklearmedizin.

[14] Wagenknecht G (2013). MRI for attenuation correction in PET: methods and challenges. MAGMA.

[15] Eiber M (2011). Value of a Dixon-based MR/PET attenuation correction sequence for the localization and evaluation of PET-positive lesions. Eur J Nuc Med Mol Ima.

[16] Beyer T (2013). Nuclear medicine 2013: from status quo to status go. Eur J Nucl Med Mol Imaging.

[17] Afshar-Oromieh A (2014). Comparison of PET/CT and PET/MRI hybrid systems using a 68Ga-labelled PSMA ligand for the diagnosis of recurrent prostate cancer: initial experience. Eur J Nucl Med Mol Imaging.

[18] Wagner H (1999). Fused image tomography: where do we go from here?. J Nucl Med.

[19] http://www.esor.org/cms/website.php?id=/en/programmes/esor_courses_for_edir/hybrid_imaging.htm.

[20] Krippendorff K (2013). Content analysis. An introduction to its methodology.

[21] Delaloye AB (2007). White paper of the European Association of Nuclear Medicine (EANM) and the European Society of Radiology (ESR) on multimodality imaging. Eur J Nucl Med Mol Imaging.

[22] Gourtsoyiannis N (2007). White paper of the European Society of Radiology (ESR) and the European Association of Nuclear Medicine (EANM) on multimodality imaging. Eur Radiol.

[23] (ESR), E.A.o.N.M.E.E.S.o.R (2012). Multimodality imaging training curriculum--parts II and III. Eur J Nuc Med Mol Ima.

[24] Frey K (2011). ABNM position statement: nuclear medicine professional competency and scope of practice. J Nucl Med.

[25] Delbeke D (2012). SNMMI/ABNM joint position statement on optimizing training in nuclear medicine in the era of hybrid imaging. J Nucl Med.

[26] Stegger L (2008). EANM-ESR white paper on multimodality imaging. Eur J Nuc Med Mol Ima.

[27] http://www.bundesaerztekammer.de/aerztetag/beschlussprotokolle-ab-1996/113-daet-2010/top-iii/neue-bezeichnungen/58-nuklearmedizinische-diagnostik-in-der-radiologie/.

[28] http://www.bundesaerztekammer.de/aerztetag/beschlussprotokolle-ab-1996/113-daet-2010/top-iii/neue-bezeichnungen/59-roentgendiagnostik-in-der-nuklearmedizin/.

[29] https://en.wikipedia.org/wiki/Habitus_(sociology). Accessed 17 Apr 2018.

[30] Bourdieu P, Loïc J. An invitation to reflexive sociology. Chicago: The University of Chicago Press; 1992.

[31] Howard SA (2015). Cancer imaging training in the 21st century: an overview of where we are, and where we need to be. J Am Coll Radiol.

[32] Manfreda K (2008). Web surveys versus other survey modes: a meta-analysis comparing response rates. Int J Market Res.

[33] Cook C, Heath F, Thompson R (2000). A meta-analysis of response rates in web- or internet-based surveys. Educ Psychol Meas.

